# Long-Standing Lambert–Eaton Myasthenic Syndrome Caused by Undetectable Small-Cell Lung Cancer: Why We Should Follow-Up LEMS

**DOI:** 10.3390/diagnostics12071542

**Published:** 2022-06-24

**Authors:** Bo Young Hong, Ho Jung An, Seong Hoon Lim

**Affiliations:** 1 Department of Rehabilitation Medicine, St Vincent’s Hospital, College of Medicine, The Catholic University of Korea, Seoul 06591, Korea; byhong@songeui.ac.kr; 2 Division of Medical Oncology, Department of Internal Medicine, St. Vincent’s Hospital, College of Medicine, The Catholic University of Korea, Seoul 06591, Korea; meicy@catholic.ac.kr

**Keywords:** lung cancer, small cell carcinoma, small cell lung cancer, paraneoplastic syndrome, Lambert–Eaton myasthenic syndrome, LEMS

## Abstract

Physicians often encounter patients with unexplained muscle weakness and dysphagia. Lambert–Eaton myasthenic syndrome (LEMS) can cause unexplained weakness or dysphagia and is often accompanied by neoplastic conditions. A 64-year-old man who had several risk factors—14 kg weight loss over the last 4 years, 20 years of experience working as a coal miner, and being a 50 pack-year ex-smoker—complained of dysphagia, intermittent diplopia, mild weakness, and hypotonia. The initial computed tomography (CT) and follow-up positron emission tomography (PET) CT did not reveal any malignancy. After continuous follow-up for this LEMS, small-cell lung cancer (SCLC, cTxN1M0) was found on a serial follow-up chest CT 21 months after the LEMS diagnosis. The patient was treated with chemotherapy. LEMS is rare and is often accompanied by malignancy. This case highlights the importance of being concerned about LEMS diagnoses and of long-term follow-up for unexplained LEMS.

Physicians often encounter patients with serious unexplained neurological symptoms. If the physician follows up on the neurological findings, diseases related to paraneoplastic syndrome may be uncovered. Lambert–Eaton myasthenic syndrome (LEMS) is characterized by common oculobulbar symptoms, a good response to edrophonium, areflexia, and typical findings on the repetitive nerve stimulation (RNS) test [1]. More than half of LEMS is associated with malignancy, mostly small-cell lung cancer (SCLC) [1,2,3]. When encountering patients with LEMS, a physician should decide how long to trace the disease in a patient when no culprit lesion, such as lung cancer, is initially found. In this paper, we present images and a brief summary of a patient who had many risk factors for lung cancer and was diagnosed with LEMS. We were concerned about the diagnosis and therefore continuously followed up with the patient. Finally, a serial chest computed tomography (CT) revealed SCLC (cTxN1M0) 21 months after the diagnosis of LEMS. Considering the increasing prevalence of lung cancer, this case highlights the importance of serial, long-term follow-up for unexplainable diseases, which may be related to paraneoplastic syndrome.

A 64-year-old man visited the department of rehabilitation complaining of dysphagia and generalized limb weakness. Several factors could be related to his malignancy: 14 kg weight loss over the last 4 years, 20 years of experience working as a coal miner, and being a 50 pack-year ex-smoker. The initial neurological examination showed intermittent diplopia, mild weakness, hypotonia of all limbs, no pathologic reflexes, and no fasciculation of the tongue or limb muscles (Table 1).

Low-frequency RNS revealed 40% decrements in the abductor digiti minimi and trapezius muscles, and post-exercise facilitation. Rapid RNS showed facilitation in the abductor digiti minimi. Other needle electromyographic tests did not show any other abnormalities, such as denervation potentials, neuromyotonia, or myotonic discharges [4]. We diagnosed LEMS of unknown origin, prescribed pyridostigmine and low-dose oral corticosteroids, and recommended serial chest CT follow-up to uncover any hidden malignancies (Figure 1A). The serum acetylcholine receptor antibody was negative, as were paraneoplastic antibodies such as Hu, Ri, Yo, and aquaporin-4 autoantibodies. The response to pyridostigmine was positive. An initial chest CT did not show malignancy or thymoma. Nine months later, the follow-up CT and positron emission tomography (PET) CT for malignancy were negative (Figure 1B,C). Twenty-one months after the diagnosis of LEMS, we found an enlarged lymph node in the right interlobar nodal station, suggesting small-cell carcinoma, and thus diagnosed him with SCLC (cTxN1M0, Figure 1D). The patient was treated with chemotherapy. Most of his symptoms related to LEMS were reduced, although mild weakness and dysphagia have persisted.

An initial presentation of LEMS, induced by paraneoplastic syndrome in SCLC, is rare [2,5]. In this case, we discovered SCLC 21 months after the initial diagnosis of LEMS. The accompanying LEMS is important and can be an indicator of a good prognosis of SCLC [3]. In this case, we followed the LEMS for 21 months and detected cTxN1M0 SCLC. The patient was treated and has a favorable prognosis.

A recent study proposed a lung cancer prediction model in patients with LEMS, which consists of six risk factors: bulbar/neck weakness, male sexual impotence, weight loss ≥ 5%, tobacco use at onset, age ≥ 50 years, and Karnofsky performance < 70 [6]. Our patient had three of these factors: bulbar weakness, weight loss ≥ 5%, and age ≥ 50 years. This gave him an estimated 60% probability of having SCLC. Considering his occupation and history of smoking, his probability of having SCLC was higher than 60%. Another report presented a patient with co-existing myasthenia gravis and LEMS-related SCLC [7]. Therefore, incidental LEMS, with or without other neuromuscular junctional disorders, suggests the possibility of SCLC, and physicians should be concerned about diagnoses of incidental LEMS.

LEMS is considered a neuromuscular autoimmune disorder associated with presynaptic voltage-gated calcium channel (VGCC) binding antibodies [8]. VGCC binding antibodies that target the alpha1A subunit of P/Q-type VGCCs are usually associated with LEMS [9,10]. However, that antibody test is not available clinically in many countries because it is not covered by insurance or for other reasons. The test for VGCC binding antibodies is not available in our country (the Republic of Korea). The other antibody tests performed showed negative results, including for the acetylcholine receptor, anti-Hu, anti-Ri, anti-Yo, and anti-aquaporin-4 antibodies.

One limitation of our report was the inability to perform certain laboratory tests. However, considering the global medical environment, this limitation reflects the actual clinical environment in many countries.

LEMS is rare and is often accompanied by malignancy. This case highlights the importance of being concerned about LEMS diagnoses and of long-term follow-ups for unexplained LEMS.

## Figures and Tables

**Figure 1:**
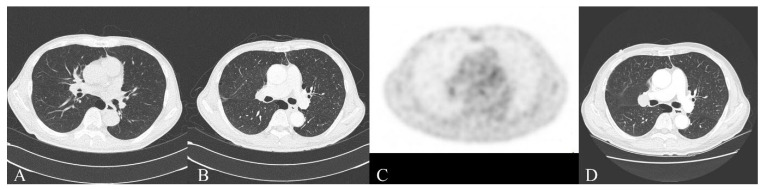
The first CT showed emphysema, and the final CT showed an enlarged LN in the right interlobar nodal station. (**A**) Chest CT at the time of diagnosis with LEMS; (**B**) chest CT 9 months after the diagnosis of LEMS; (**C**) PET CT 9 months after the diagnosis of LEMS; and (**D**) chest CT 21 months after the diagnosis of LEMS, allowing for a diagnosis of lung cancer.

**Table 1:** A clinical and electrophysiological summary of the patient at initial presentation.

Category	Subtype	Patient Findings
Clinical findings	Risk factor	20 years of experience working as a coal miner, 50 pack-year ex-smoker
	Presenting symptoms	Dysphagia, generalized limb weakness, 14 kg weight loss over the last 4 years
	Neurologic abnormality	Intermittent diplopia, hypotonia of all limbs
Electrodiagnostic findings	Repetitive nerve stimulation	Low-frequency repetitive nerve stimulation revealed 40% decrements Rapid repetitive nerve stimulation showed facilitation
Laboratory tests		Negative
Chest CT		Negative
